# Physical processing for decellularized nerve xenograft in peripheral nerve regeneration

**DOI:** 10.3389/fbioe.2023.1217067

**Published:** 2023-05-30

**Authors:** Ming-Wei Hsu, Szu-Han Chen, Wan-Ling Tseng, Kuo-Shu Hung, Tzu-Chun Chung, Sheng-Che Lin, Jahyun Koo, Yuan-Yu Hsueh

**Affiliations:** ^1^ Division of Plastic and Reconstructive Surgery, Department of Surgery, National Cheng Kung University Hospital, College of Medicine, National Cheng Kung University, Tainan, Taiwan; ^2^ Center of Cell Therapy, National Cheng Kung University Hospital, College of Medicine, National Cheng Kung University, Tainan, Taiwan; ^3^ International Research Center for Wound Repair and Regeneration, National Cheng Kung University, Tainan, Taiwan; ^4^ Division of Plastic and Reconstructive Surgery, Department of Surgery, Tainan Hospital, Ministry of Health and Welfare, Tainan, Taiwan; ^5^ Department of Orthopedic Surgery, E-Da Hospital, Kaohsiung, Taiwan; ^6^ Division of Plastic Surgery, Department of Surgery, An-Nan Hospital, China Medical University, Tainan, Taiwan; ^7^ School of Biomedical Engineering, Korea University, Seoul, Republic of Korea

**Keywords:** decellularized nerve xenograft, peripheral nerve regeneration, physical processing, freeze-thaw, perfusion, immersion and agitation, sonication, supercritical fluids

## Abstract

In severe or complex cases of peripheral nerve injuries, autologous nerve grafts are the gold standard yielding promising results, but limited availability and donor site morbidity are some of its disadvantages. Although biological or synthetic substitutes are commonly used, clinical outcomes are inconsistent. Biomimetic alternatives derived from allogenic or xenogenic sources offer an attractive off-the-shelf supply, and the key to successful peripheral nerve regeneration focuses on an effective decellularization process. In addition to chemical and enzymatic decellularization protocols, physical processes might offer identical efficiency. In this comprehensive minireview, we summarize recent advances in the physical methods for decellularized nerve xenograft, focusing on the effects of cellular debris clearance and stability of the native architecture of a xenograft. Furthermore, we compare and summarize the advantages and disadvantages, indicating the future challenges and opportunities in developing multidisciplinary processes for decellularized nerve xenograft.

## 1 Introduction

The current gold standard for peripheral nerve repair in segmental defects is autologous nerve grafting (Kalomiri et al., 1994). However, several limitations hamper the clinical practice, such as limited supply, donor site morbidity, and size discrepancy (Hu et al., 2009). Alternative autologous nerve substitutes, such as autologous veins, muscles, and tendons have been utilized with variable outcomes (Ray et al., 2011). Because of the abovementioned drawbacks, tissue engineered nerve grafts (TENG) were developed, aiming to provide a biomimetic scaffold for peripheral nerve regeneration (Houschyar et al., 2016). Although the Food and Drug Administration had approved several off-the-shelf synthetic nerve conduits on the market, but most of them are limited to nerve gaps <3 cm or <0.5 cm in small or large diameter nerves, respectively (de Ruiter et al., 2009). In addition, a recent review does not support the use of currently available TENG over standard nerve repair (Thomson et al., 2022). To achieve a better outcome in longer nerve gaps, nerve allografts are harvested and processed under a commercial decellularization process. Avance^®^, a mature commercial product which is produced by processing human nerve tissue with a combination of detergent decellularization, chondroitinase CSPG degradation, and gamma-irradiation sterilization, has clinical evidence to overcome up to 70 mm nerve gap in sensory, mixed, and motor nerve repair (Gunn et al., 2010; Safa et al., 2020). However, limited donor sources, high costs, low temperature preservation, and potential immune rejection still remain as clinical concerns for its wide usage.

Xenotransplantation was developed due to the unlimited availability of sources. In general, fresh xenografts elicit an immune response that causes graft rejection. The presence of non-self-antigenic epitopes triggers the activation of T- and B-lymphocytes. This, in turn, leads to the activation of an immune response mediated by antibodies, ultimately resulting in the rejection of these cells. (Fox et al., 2001; Vadori and Cozzi, 2014; Lopresti et al., 2015). Despite using immunosuppressive drugs, decellularization is an effective approach to utilize the xenogeneic and allogeneic tissues. The cellular components of tissues are the main cause of an adverse host response. To mitigate immune rejection, several decellularization techniques were developed to remove cellular components to reduce immunogenic reactions while preserving native scaffold or extracellular matrix (ECM) microstructure (Rana et al., 2017). Several recent studies reported that acellular nerve xenografts have similar effects on regeneration and immunocompatibility compared with acellular nerve allografts (Zhang et al., 2010; Huang et al., 2015). The concept of Decellularized extracellular matrix (dECM) is established as using various methods (physical, enzymatic, or chemical) to lyse cells and remove the intracellular components from a tissue while preserving the native extracellular components and the cues for cell proliferation and differentiation (Dahl et al., 2003; Lopresti et al., 2015; Kim et al., 2019). Although regeneration can occur in short nerve gaps regardless of an immune response, nerve growth was suppressed in long nerve gaps (Choi and Raisman 2003).

Zhang and Chen summarized several decellularization protocols along with their mechanisms and disadvantages, including physical, chemical, and enzymatic treatments in organ or tissue (Zhang et al., 2022). For nerve growth, a biomimetic neural scaffold is crucial, and thus, limited decellularization methods are applicable. Chemical-based decellularization was widely used in nerve tissues, but the major concern was the possible interruption of nerve growth by the residual chemical agents (Han et al., 2019). Enzymatic treatments provide high specificity for the removal of cellular components; however, they cannot be removed completely that might induce severe distortion of the ECM structure.

**FIGURE 1:**
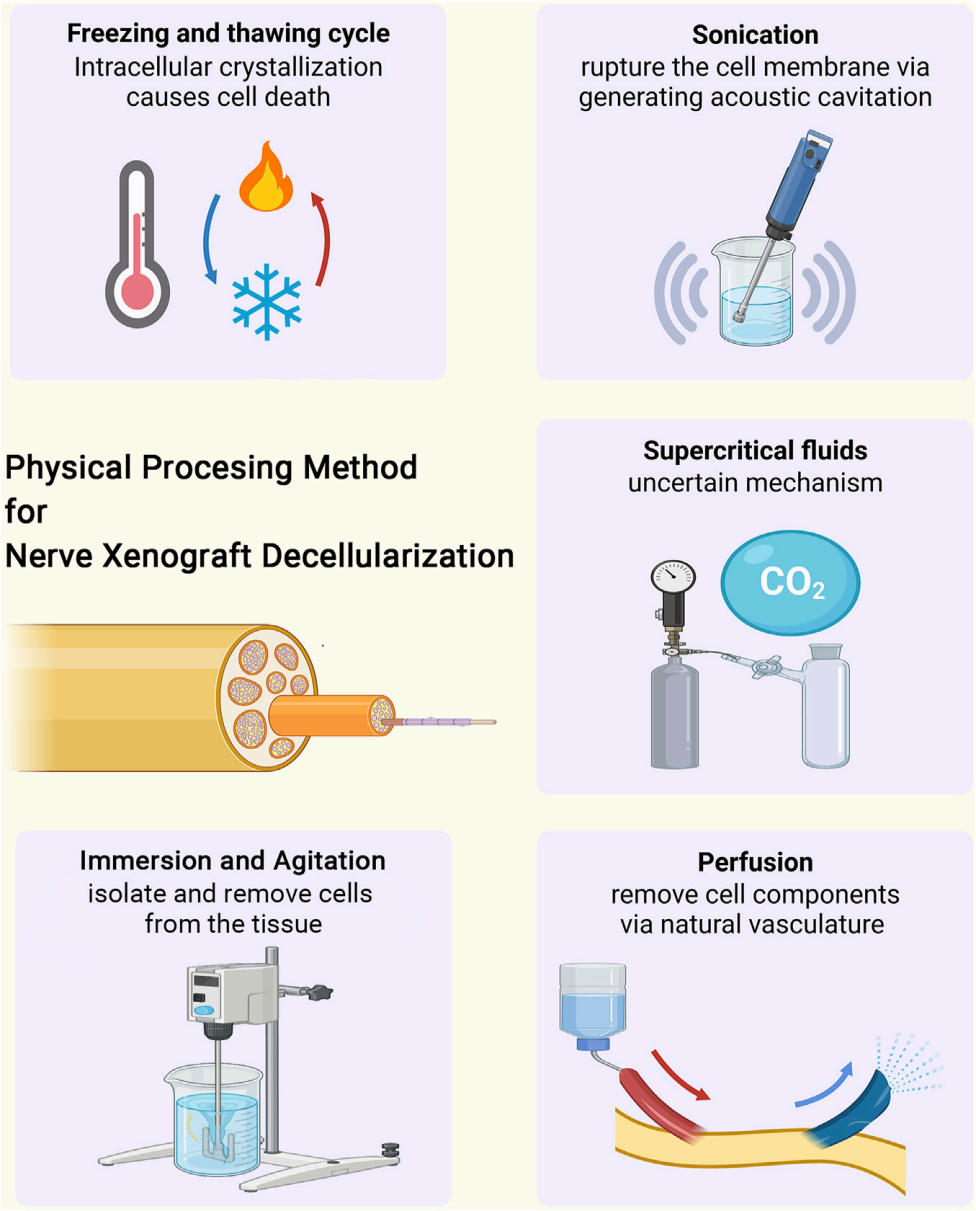
The physical processing methods for tissue decellularization of nerve xenograft, including freezing and thawing cycle, sonication, immersion and agitation, perfusion and supercritical fluids.

This minireview aims to focus on the latest five physical-based decellularization methods for peripheral nerve xenotransplantation (Figure 1). Table 1 summarizes the mechanisms, advantages, and disadvantages of the current physical processing methods.

**TABLE 1:** Summary of current physical processing methods for decellurized nerve xenograft.

Method	Mechanism	Xenograft source	Advantages	Disadvantages	References
Freeze-thaw cycles	Intracellular crystallization causes cell death	porcine, rabbit, dog	easy, low-cost	limited ability to eliminate cells	Ide (1983), Ide, Tohyama, Yokota, Nitatori, & Onodera (1983), Osawa et al. (1990), Evans et al. (1998), Hess et al. (2007), Jesuraj et al. (2014), Kaizawa et al. (2017), Philips et al. (2018)
Sonication	rupture the cell membrane via generating acoustic cavitation	rabbit	low-cost; can shorten processing time	can’t remove DNA content	Hudson et al. (2004), Boriani et al. (2017), Bolognesi et al. (2022), Suss et al. (2022)
Perfusion	Pressure induced by perfusion via natural vasculature can remove cellular components	porcine	might overcome long nerve gap	surgical complexity, donor site morbidity and limited nerve availability	Wüthrich et al. (2020)
			avoid ischemic damage and central necrosis		
Immersion and Agitation	isolate and remove cells from the tissue	rat	Easy, might overcome long nerve gap	Time consumable	Vasudevan et al. (2014)
Supercritical fluids	Uncertain	porcine	environmental friendly, nontoxicity, low cost, disinfection	low ability of defatting	Isenschmid et al. (1995), White et al. (2006), Casali et al. (2018), Topuz et al. (2020), Wei et al. (2022)

## 2 Different approaches of physical processing

This minireview aims to clarify the latest four physical-based decellularization methods for peripheral nerve xenotransplantation (Figure 1). Table 1 summarizes the mechanisms, advantages, and disadvantages of the current physical processing methods.

### 2.1 Freeze–thaw cycles

Freeze–thaw cycles refer to a repetitive freeze-drying process (Zhang et al., 2022). In nerve xenografts, the aim of cold preservation and freeze–thaw cycles is to destroy the nerve cell membranes by inducing the formation of intracellular ice crystals, thereby reducing the immunogenicity of nerve xenografts (Hare et al., 1993; Fox et al., 2005; Philips et al., 2018). Cold preservation and freeze–thaw cycles are easy to manipulate and an initial step in many decellularization protocols, as the protocols can be adjusted according to nerve length and diameter, depending on the laboratory preferences (Lasso and Deleyto, 2017).

However, studies have shown that the ultrastructure of nerves may be damaged by the freeze–thaw cycles, although the mechanical properties of the nerves are preserved (Osawa et al., 1990; Evans et al., 1998). A slower recovery in rats that received frozen grafts compared with those that received fresh autografts in a 2 cm median-nerve-gap rat model using Beagle dog acellular frozen xenografts indicate that freezing leads to a barren microenvironment for nerve regeneration (Accioli De Vaconcellos et al., 1999). Furthermore, the freeze–thaw process led to nerve xenograft rejection caused by the residual cells and debris (Lu et al., 2009). Despite the easy manipulation of freeze-thaw cycles, they might be responsible for damage to the nerve microstructure and a depleted environment for nerve regeneration.

### 2.2 Perfusion

Perfusion refers to the process of introducing circulating agents through the intrinsic vascular system of organs or tissues. This technique is typically used in larger, thicker tissues or whole organs (Goh et al., 2018). Only one study has reported the use of perfusion decellularization in peripheral nerve repair. Wüthrich and Lese applied perfusion decellularization to surgically procured vascularized porcine sciatic nerves (Wüthrich et al., 2020). A 3D microcomputed tomography imaging showed preserved vasculature and the ECM component. The dissected graft contained more external connective tissues, and the measurable growth factors were detectable at low levels. These results suggest that the biological activity of the graft may be retained and could promote nerve regeneration. An *in vitro* study revealed the potential for re-endothelialization, but no *in vivo* study has been conducted yet. These nerve scaffolds can be created for specific lesions and are becoming increasingly available, with the potential to overcome large nerve gaps.

### 2.3 Immersion and agitation

Immersion and agitation refers to the process of submerging tissues into decellularization solutions with constant mechanical agitation (Crapo et al., 2011; Zhang et al., 2022). Compared with perfusion treatment, immersion and agitation is used in processing small, fragile and thin sections of tissues without innate vascular structures (Alshaikh et al., 2019). The efficiency of this method depends on different paraments including agitation intensity, decellularization agent and tissue dimension (Duisit et al., 2018). Immersion and agitation methods of tissue decellularization have been described for a wide variety of tissues, including peripheral nerves (Hudson et al., 2004; Karabekmez et al., 2009; Vasudevan et al., 2014). However, most of the decellularization solutions were chemical, detergent, or enzymatic solutions (Keane et al., 2015). To the best of our knowledge, only Vasudevan et al. reported an immersion and agitation method with detergent-free solution in peripheral nerve field (Vasudevan et al., 2014). In their design, the nerve grafts were immersed with detergent-free solution and were cultured at 37°C with 5% CO2 for 2 weeks under constant agitation, which was performed to initiate Wallerian degeneration *in vitro* to clear axonal and myelin debris inside the nerves. In the 3.5-cm sciatic nerve transection rat model, nerve regeneration was identical to that of detergent-processed grafts, while the functional nerve regeneration was only observed in detergent-free decellularized grafts at 12 weeks. This method did not significantly affect the ECM surface structure, collagen structure and integrity, mechanical strength, and GAG content, but may cause more damages to tissues due to the limited diffusion of chemical, detergent, or enzymatic decellularization solutions by agitation (Wilson et al., 2016; Simsa et al., 2019; Zhang et al., 2022).

### 2.4 Sonication

Sonication is a method of rupturing the cell membrane by generating acoustic cavitation bubbles and inducing shear stress effect. It can assist in the penetration of agents by vibration as well as remove cellular debris. It was demonstrated that coupling freeze–thawing with sonication contributes to a cell-free and aseptic xenograft in a shorter time than applying freeze–thawing alone in a rabbit peripheral nerve model (Boriani et al., 2017). Based on these results, they further developed a new method of soaking the nerve tissues in decellularizing solutions, with combination of sonication and freeze–thaw cycles (Bolognesi et al., 2022). This new method was validated through histology and immunohistochemistry, showing its application to decellularized xenografts with similar or better results compared with the Hudson technique (Hudson et al., 2004). However, it was observed that sonication during chemical decellularization did not remove deoxyribonucleic acid (DNA) content but only cellular debris and myelin sheaths (Suss et al., 2022). So far, no study has utilized sonication as a single process for xenograft decellularization.

### 2.5 Supercritical fluids

Supercritical fluids are fluids above their critical pressure and temperature that possess characteristics such as low viscosity and high diffusivity. Supercritical carbon dioxide (ScCO_2_) is an environmentally friendly solvent that is widely used in the field of biomedicine and biomaterials due to its nontoxicity, low cost, and superior disinfection and sterilization abilities (Subramaniam et al., 1997; Casali et al., 2018). It has a critical pressure 7.38 MPa and a critical temperature 31°C. ScCO_2_ has been shown to remove cells from the tissues while maintaining the ECM structure. However, the exact mechanism by which this occurs is unclear. The hypothesis that high pressure induces cell bursting, as claimed by Topuz et al., has been refuted (Isenschmid et al., 1995; White et al., 2006). It is hypothesized that ScCO2 might induce hypoxia, which has been validated by histological and morphological analyses after successful decellurization of bovine optic nerves using ScCO2 (Topuz et al., 2020). Wei et al. developed a porcine acellular nerve xenograft based on supercritical extraction technology and validated it in a 15-mm rat sciatic nerve model (Wei et al., 2022). A porous nerve basement membrane with a well-preserved 3D structure was observed. Low cytotoxicity was noted *in vitro*, leading to decreased immune response *in vivo*. The ScCO_2_ treatment group was found to be similar to the autologous nerves in terms of regenerated nerve quality, target muscle wet weight regain, and motor function recovery. Moreover, the hybrid detergent plus ScCO_2_ treatment demonstrated better outcomes in terms of decellularization and defatting compared to ScCO_2_ alone (Casali et al., 2018).

## 3 Challenges and opportunities

In recent decades, tissue engineering has been applied in the field of regenerative medicine to peripheral nerve repair, relying on the three main pillars: scaffolds, cells, and growth factors (Carvalho et al., 2019). In scaffolds, preserving the biomimetic microenvironment is essential, whereas in xenografts, the removal of cells is critical to prevent subsequent immune rejection. Gilpin and Yang et al. measured four aspects of the decellularized ECM to assess the quality of decellularization: removal of cells, elimination of genetic material, preservation of the protein content, and retention of the mechanical properties (Gilpin and Yang, 2017). Carpo et al. proposed specific criteria for assessing the efficacy of cell removal: the decellularized ECM must have the following: 1) less than 50 ng double-stranded DNA per mg ECM dry weight, 2) less than 200 bp DNA fragment length, and 3) no visible nuclear material by 4′,6-diamidino-2-phenylindole staining (Crapo et al., 2011).

Chemical and enzymatic approaches are the most widely applied methods for peripheral nerve decellularization, but the possible toxicity of the chemicals and destruction of the ECM proteins are major drawbacks of these methods. Physical treatments like modulating temperature or pressure have been effective, but it comes with limitations because of the natural architecture of nerves. Freeze–thawing is widely used and an important step in all decellularization protocols. However, debris retention and potential microstructure damage might restrict the *in vivo* therapeutic effects. Perfusion decellularization was developed to overcome long nerve gaps using vascularized porcine sciatic nerves, but the evidence of an *in vivo* study is lacking. Immersion and agitation decellularization is commonly used but generally with chemical, detergent, and/or enzymatic solutions. Sonication is integrated with other methods to assist in removing cellular debris. However, the clearance of DNA content is questionable. Supercritical fluids show promise with advantages of nontoxicity, low cost, superior disinfection and sterilization abilities, and can solely and effectively remove cells. Pure physical treatment has a limited but acceptable effect in decellularization compared with chemical and enzymatic approaches. Both immersion and agitation and supercritical fluids had shown that identical decellularization efficacy can be achieved as compared to chemical or enzymatic approaches. Further investigations are required to validate the *in vivo* therapeutic outcomes. With comprehensive understanding of the physical processing methods, multidisciplinary integration of different approaches are expected to elicit accumulative benefits, in terms of reducing immunogenicity and preserving the mechanical properties and microenvironment of native nerve tissue.
